# Spaceflight modulates gene expression in the whole blood of astronauts

**DOI:** 10.1038/npjmgrav.2016.39

**Published:** 2016-12-08

**Authors:** Jennifer Barrila, C Mark Ott, Carly LeBlanc, Satish K Mehta, Aurélie Crabbé, Phillip Stafford, Duane L Pierson, Cheryl A Nickerson

**Affiliations:** 1 Center for Infectious Diseases and Vaccinology, The Biodesign Institute, Arizona State University, Tempe, AZ, USA; 2 Biomedical Research and Environmental Sciences Division, NASA Johnson Space Center, Houston, TX, USA; 3 Department of Microbiology and Immunology, Program in Molecular Pathogenesis and Immunity, Tulane University Health Sciences Center, New Orleans, LA, USA; 4 EASI/Wyle Laboratories, Houston, TX, USA; 5 Center for Innovations in Medicine, The Biodesign Institute, Arizona State University, Tempe, AZ, USA; 6 School of Life Sciences, Arizona State University, Tempe, AZ, USA

## Abstract

Astronauts are exposed to a unique combination of stressors during spaceflight, which leads to alterations in their physiology and potentially increases their susceptibility to disease, including infectious diseases. To evaluate the potential impact of the spaceflight environment on the regulation of molecular pathways mediating cellular stress responses, we performed a first-of-its-kind pilot study to assess spaceflight-related gene-expression changes in the whole blood of astronauts. Using an array comprised of 234 well-characterized stress-response genes, we profiled transcriptomic changes in six astronauts (four men and two women) from blood preserved before and immediately following the spaceflight. Differentially regulated transcripts included those important for DNA repair, oxidative stress, and protein folding/degradation, including *HSP90AB1*, *HSP27*, *GPX1*, *XRCC1*, *BAG-1*, *HHR23A*, *FAP48*, and *C-FOS*. No gender-specific differences or relationship to number of missions flown was observed. This study provides a first assessment of transcriptomic changes occurring in the whole blood of astronauts in response to spaceflight.

During spaceflight, astronauts experience a variety of physical and psychological stressors, including acceleration at launch, isolation, confinement, high levels of ionizing radiation, sleep deprivation, and microgravity, which have been linked to a variety of physiological changes, including dampened immune function, bone and muscle loss, and increased viral reactivation.^1–5^ The molecular basis of spaceflight-induced responses remains unclear and may provide critical insights into health outcomes associated with space travel. Targeted spaceflight-associated changes in cytokine and antibody expression have been previously reported for astronauts,^1–3^ and more recently Terada *et al* reported the effects of spaceflight on hair follicle gene expression in astronauts.^6^ To evaluate the potential impact of the spaceflight environment on molecular pathways mediating cellular stress responses, we performed a first-of-its-kind pilot study to assess spaceflight-related changes in the expression of stress-response genes in the whole blood of astronauts in response to spaceflight.

Transcriptional profiling of 234 well-characterized stress-response genes was performed using total RNA isolated from whole blood obtained from six consenting astronauts 10 days before launch aboard the space shuttle and 2–3 h after return to Earth (see Supplemental Methods). Four men between the ages of 38 and 47 (mean=43 years) and two women between the ages of 38 and 44 (mean=41 years) participated in the study. Each of these astronauts flew aboard one of four space shuttle missions ranging between 10 and 13 days duration that took place during a 2-year period. Previously published studies using blood and saliva samples from these same crew members before, during and just after spaceflight indicated that all six astronauts displayed increases in Epstein Barr virus reactivation both during and immediately post flight, which is consistent with these individuals being in a stressed state.^4,5^

Microarray analysis revealed some variation in the gene-expression patterns displayed across individual crew members. This is not surprising given the smaller study size due to the exceptionally rare opportunity for this type of sample collection, as well as other studies that have shown that there can be a wide range of individual and temporal variability in gene-expression patterns in human blood.^7^ High variability was also observed in gene-expression studies using astronaut hair follicles.^6^ We found six transcripts that displayed significant (*P*<0.05) changes in expression in the crew in response to spaceflight when compared with pre-flight levels (Table 1). Two additional differentially regulated transcripts were identified following outlier removal. No gender-specific differences or relationship to number of missions flown were observed. It is important to note that as for many human spaceflight studies, the small number of human subjects available for analysis is a limiting factor to the statistical power. False discovery rate algorithms determined no significant genes were expressed, likely due to these low study group numbers. The NASA Twins Study with astronauts Scott Kelly and Mark Kelly performed during the One-Year Mission may face similar challenges in their gene-expression data interpretation, as there is an *n* of 1 in each condition (spaceflight and ground). However, one advantage in the Twins Study over the present one is the longitudinal sample collection that could be performed during the year that Kelly spent in space. It will be intriguing to compare the results from gene-expression analyses between the two studies.

Genes altered in expression encode proteins of known importance for DNA repair (*XRCC1 *and* HHR23A*), oxidative stress (*GPX1*), and chaperones which have key roles in protein folding and/or proteasomal degradation (*HSP27 *and *HSP90AB1*). The finding that genes encoding DNA-repair proteins are downregulated supports previous studies which showed post-flight increases in chromosomal aberrations in lymphocytes of astronauts^8^ and indications of increased DNA damage in astronauts after long-duration spaceflight.^9^ Kumari *et al* previously reported that exposure of human lymphocytes to simulated microgravity over the course of 7 days led to increased DNA damage.^10^ This damage was accompanied by progressive decreases in the expression of a number of representative DNA-repair genes, leading the authors to postulate that impaired DNA-repair capacity could lead to increased damage and mutations. In a separate study, it was reported that 1 week of hindlimb unloading in male BALB/c mice led to the downregulation of multiple DNA-repair genes including ERCC 1, ERCC 3, ERCC5, ERCC6, and XPC in the testes of BALB/c mice.^11^

Of particular interest was the downregulation of *GPX1,* which encodes for glutathione peroxidase (GPX1), an enzyme that protects cells from oxidative damage, modulates the immune response (including delayed type hypersensitivity/DTH), and has been associated with increased viral titers in herpes viral infections.^12^ Given that spaceflight depresses the DTH response,^2^ alters cellular oxidative functions,^9,13–17^ and increases herpes viral reactivation in astronauts on orbit,^4,5^ additional detailed studies related to glutathione and oxidative stress may fundamentally advance our understanding of mechanisms underlying risks to crew health during a mission. Several previous studies have indicated the potential for alterations in oxidative stress and antioxidant defense status in response to spaceflight and spaceflight-analog models in humans and animals.^9,13–16^ Although the reported magnitude and direction of the expression/activity levels of specific redox enzymes (including GPX1) were variable across these studies (likely due to differences in experimental parameters such as the use of human versus animal subjects, mission duration, etc) the overall findings support the potential for increased oxidative stress and/or decreased antioxidant defense capacity in response to spaceflight. It is also interesting to note that GPX1 is a selenoprotein and decreased serum selenium levels have been reported in astronauts post landing as compared with pre-launch.^9^ Therefore, additional insight into GPX1 and other oxidative defense mechanisms may serve as a guiding principle for establishing nutritional requirements to ensure health safety and performance of the crew during human exploration of space.^9,18^ When we compared these transcriptomic findings from blood samples to a previously published study using hair samples,^6^ we did not find similarities in the genes that were differentially regulated pre- and post flight, which could be due to a variety of experimental differences, including sample source (whole blood versus hair follicles) and mission duration (10–13 days in the present study compared with ~6 months in the study by Terada *et al*^6^).

To our knowledge, this study represents the first report of transcriptomic changes occurring in the blood of astronauts in response to spaceflight. Several transcripts encoding stress-response genes were suppressed in the crew after exposure to the microgravity environment, including those important for DNA repair, oxidative stress, detoxification, and protein folding/degradation. These processes are vital for maintaining human health by mediating cellular pathways that serve to protect against both environmental and physiological stressors and have been implicated in a broad spectrum of diseases. Since changes in gene expression in peripheral blood may be attributed to a number of factors, including changes in the distribution of blood cellular subsets (which has been detected post flight in the crew),^1–3^ future studies designed to establish direct links between specific physiological/cellular adaptations underlying these transcriptomic changes in response to the microgravity environment will be important. It is important to note that no direct correlation can be made between gene-expression changes observed in this study and the disease status or potential for the development of disease in these individuals. This study provides an initial foundation into the molecular genetic response profiles of astronauts during spaceflight from which additional research into alterations in crew health and performance can be investigated. This kind of research could lead to human health countermeasures to mitigate risk during the transition from short-to-long-duration flight and could potentially translate to health benefits for the general public.

## Figures and Tables

**Table 1:** Differentially regulated genes in astronaut blood in response to spaceflight

*Gene*	*Name/description*	*Ratio post/pre*	P* value*
*Downregulated genes*			
*HSP27*	27 kDa heat-shock protein (HSP27); stress-responsive protein 27 (SRP27); estrogen-regulated 24 kDa protein; heat shock protein family B (small) member 1 (HSPB1)	0.44	0.045
*BAG-1*	BCL-2-binding athanogene-1; glucocorticoid receptor-associated protein RAP46	0.37	0.015
*XRCC1*	DNA repair protein – X-ray repair cross-complementing factor 1	0.45	0.044
*HSP90AB1*	90 kDa heat-shock protein-β; 84 kDa heat-shock protein-β (HSP84); heat shock protein 90 class B (HSPCB)	0.41	0.023
*GPX1*	Glutathione peroxidase (GSHPX1; GPX1)	0.52	0.041
*HHR23A*	UV excision repair protein—human homolog of *Saccharomyces cerevisiae* RAD23A	0.53	0.003^a^
*FAP48*	48 kDa FK506 Binding Protein (FKPB)-associated protein	0.55	0.022^b^
			
*Upregulated genes*			
*C-FOS*	c-Fos proto-oncogene	3.11	0.021

Abbreviation: UV, ultraviolet.

a An outlier was identified for the pre-flight mean. Excluding this outlier yielded a significant *P* value. Inclusion of outlier yielded a *P* value of 0.105.

b An outlier was identified for the pre-flight and post-flight means. Excluding outliers yielded a significant *P *value. Inclusion of outliers yielded a *P *value of 0.111.
