# Supplementary material for: Spaceflight modulates gene expression in the whole blood of astronauts
Source: NPJ Microgravity. 2016 Dec 8;2:16039–. doi: 10.1038/npjmgrav.2016.39 (PMC5515525; doi:10.1038/npjmgrav.2016.39)
Supplement: Supplementary Information [file npjmgrav201639-s1.doc]

**Supplemental Methods**

## Blood collection and storage conditions

Permission to collect blood from National Aeronautics and Space Administration (NASA) astronauts was obtained from the Johnson Space Center Committee for the Protection of Human Subjects. Consenting astronauts from several shuttle missions provided whole blood samples 10 days prior to shuttle launch (L-10) and several hours after return to earth (R+0). Whole blood was collected into a Vacutainer Blood collection tubes and then transferred into PAXgene tubes (PreAnalytiX, Hombrechtikon, CH), according to the manufacturer’s instructions. The PAXgene tubes contain a proprietary mixture of chemicals that lyses blood cells, rapidly stabilizes cellular RNA, and removes protein contaminants that interfere with RNA purity. Use of the PAX gene system for this application provided total RNA that was of high purity and integrity, and demonstrated excellent performance in microarray hybridization and signal analyses. Two separate 2.5 ml samples were collected from crewmembers in order to obtain enough RNA for processing for microarrays.

## Isolation of total RNA from human whole blood and preparation for arrays

Total RNA was isolated from whole blood samples using the PAXgene Blood RNA Kit (PreAnalytiX, Hombrechtikon), according to the manufacturer’s instructions. Duplicate RNA samples were combined and DNase treated using a DNA-free kit (Ambion). RNA was quantitated by ultraviolet absorbance, concentrated by ethanol precipitation and re-suspended in deionized water (BD Biosciences Clontech) to yield a 4 μg sample of RNA for each astronaut at L-10 and R+0. RNA integrity was confirmed using denaturing formaldehyde agarose gel electrophoresis.

*cDNA expression arrays*

Expression profiles for pre-flight (L-10) and post-flight (R+0) crew samples were analyzed using the Atlas cDNA Human Stress Array (BD Biosciences Clontech, catalog # 7747-1). These application-targeted nylon arrays carry known human genes grouped into functional classes to generate highly specific and informative results through the use of a comprehensive set of well-characterized genes known to be involved in human cellular stress responses. A mixture of gene specific primers for probe synthesis reduces probe complexity, thus there is a significant increase in sensitivity with a concomitant reduction in nonspecific background. These arrays allowed for the analysis of 234 well-characterized stress response genes simultaneously and contain three negative controls to confirm specificity and nine housekeeping genes for normalizing multiple arrays.

## Probe Synthesis and Hybridization

Total RNA was converted to ^32^P-labeled first-strand cDNA by reverse transcription. Reaction volumes were optimized for increased detection on the arrays as follows: 4 μg of total RNA was combined with 2 μl gene-specific CDS primer mix (BD Biosciences Clontech). A master mix was prepared at room temperature including 4 μl 5X Reaction Buffer, 2 μl 10X dNTP Mix (for dATP label), 5 μl [α-^32^P]dATP (10uCi/ul), and 1 μl 100mM DTT. Two microliters of MMLV Reverse Transcriptase was added to the master mix, and was combined with the RNA, primer mix. All incubations were performed according to the manufacturer’s instructions. Labeled probes were purified using column chromatography (BD Biosciences Clontech). Radioactive cDNA probes were hybridized to the microarrays at 68°C overnight in ExpressHyb solution (BD Biosciences Clontech) and washed according to the manufacturer’s instructions. Arrays were exposed to a phosphoimager screen at room temperature for 3 days.

## Array Analysis

Arrays were analyzed using Atlas Image 2.0 software (Clontech Laboratories, Inc., Palo Alto, CA), according to the manufacturer’s instructions. Arrays were normalized to the following housekeeping genes: ubiquitin C (UBC), liver glyceraldehyde 3-phosphate dehydrogenase (GAPDH), tubulin alpha 1 (TUBA1), and major histocompatability complex class I C (HLAC). After background subtraction for each array, the ratio of the post-flight adjusted intensity values (R+0) to the pre-flight adjusted intensity values (L-10) was calculated. Only genes with at least four data points pre-flight and post-flight were taken into consideration for data analysis. Since paired values were not available for all data points (values below threshold due to quality control), an unpaired student t-test on intensity values was utilized for determination of significance. Differences in gene expression were considered significant when p < 0.05 and fold-change > 1.5. All current False Discovery Rate algorithms determined no significant genes expressed, but with the low number of precious samples, it was decided to proceed using standard significance testing plus the fold-change filter. Data was not log_10_ transformed, because the Kolmolgorov-Schmirnov Goodness of Fit test did not reveal that log_10_ transformed data was closer to normally distributed than untransformed data. In order to include differentially expressed genes that were not significant due to the presence of a biological outlier, outliers were identified by determining the interquartile range. Expression values that were more than 1.5 times outside the interquartile range were designated as outliers and removed for statistical analysis, which allowed for the identification of two additional differentially expressed genes (Table 1). Data submitted to GEO for platform GPL140 contain genes that have been pre-filtered by the analytical software to remove values of low certainty, resulting in missing values for some samples. Unfortunately, the original raw data in its entirety is no longer available due to physical damage at Tulane University during hurricane Katrina, but the processed values were retained in redundant locations and these were uploaded to GEO (Accession no. GSE47126).
